# Genome-Wide Transcriptional Profiling Reveals Two Distinct Outcomes in Central Nervous System Infections of Rabies Virus

**DOI:** 10.3389/fmicb.2016.00751

**Published:** 2016-05-19

**Authors:** Daiting Zhang, Feilong He, Shuilian Bi, Huixia Guo, Baoshi Zhang, Fan Wu, Jiaqi Liang, Youtian Yang, Qin Tian, Chunmei Ju, Huiying Fan, Jinding Chen, Xiaofeng Guo, Yongwen Luo

**Affiliations:** ^1^College of Veterinary Medicine, South China Agricultural UniversityGuangzhou, China; ^2^Key Laboratory of Zoonosis Prevention and Control of Guangdong Province, South China Agricultural UniversityGuangzhou, China; ^3^School of Food Science, Guangdong Pharmaceutical UniversityZhongshan, China

**Keywords:** rabies virus, transcriptome, pathogenic and attenuated strains, innate immunity, pathogenesis, MHC class II

## Abstract

Rabies remains a major public health concern in many developing countries. The precise neuropathogenesis of rabies is unknown, though it is hypothesized to be due to neuronal death or dysfunction. Mice that received intranasal inoculation of an attenuated rabies virus (RABV) strain HEP-Flury exhibited subtle clinical signs, and eventually recovered, which is different from the fatal encephalitis caused by the virulent RABV strain CVS-11. To understand the neuropathogenesis of rabies and the mechanisms of viral clearance, we applied RNA sequencing (RNA-Seq) to compare the brain transcriptomes of normal mice vs. HEP-Flury or CVS-11 intranasally inoculated mice. Our results revealed that both RABV strains altered positively and negatively the expression levels of many host genes, including genes associated with innate and adaptive immunity, inflammation and cell death. It is found that HEP-Flury infection can activate the innate immunity earlier through the RIG-I/MDA-5 signaling, and the innate immunity pre-activated by HEP-Flury or Newcastle disease virus (NDV) infection can effectively prevent the CVS-11 to invade central nervous system (CNS), but fails to clear the CVS-11 after its entry into the CNS. In addition, following CVS-11 infection, genes implicated in cell adhesion, blood vessel morphogenesis and coagulation were mainly up-regulated, while the genes involved in synaptic transmission and ion transport were significantly down-regulated. On the other hand, several genes involved in the MHC class II-mediated antigen presentation pathway were activated to a greater extent after the HEP-Flury infection as compared with the CVS-11 infection suggesting that the collaboration of CD4^+^ T cells and MHC class II-mediated antigen presentation is critical for the clearance of attenuated RABV from the CNS. The differentially regulated genes reported here are likely to include potential therapeutic targets for expanding the post-exposure treatment window for RABV infection.

## Introduction

Rabies is an acute as well as progressive encephalomyelitis caused by a neurotropic virus known as rabies virus (RABV), which is a non-segmented negative-stranded RNA virus in the genus *Lyssavirus*, family *Rhabdoviridae* (Schnell et al., 2010). Regardless of the viral variants found throughout the world, all lyssaviruses can cause rabies, resulting in the post-exposure prophylaxis (PEP) in tens of millions of humans, and more than 60,000 human deaths each year (Fooks et al., 2014). The RABV enters the nervous system through the neuromuscular junctions or nerve spindles (Lafon, 2005). In the early stages of infection, while RABV travels through peripheral nerve axons to the central nervous system (CNS), there is no clear clinical evidence of the infection. The definitive signs and symptoms of the disease do not appear until the virus reaches the CNS, when a wide range of non-specific physiological, as well as more pathognomonic signs begin to develop (Lafon, 2011).

During its migration to the CNS through the nerves, RABV has to deal with the first line of cellular defense against pathogens, which is the host's innate immune response (Chopy et al., 2011b). RABV infection activates the innate immune sensors like retinoic acid inducible gene I (RIG-I) and melanoma differentiation-associated protein 5 (MDA-5) (Wang et al., 2005; Johnson et al., 2006; Faul et al., 2010; Chopy et al., 2011a), and triggers a classical type I interferon (IFN) pathway, to create a chemoattractive environment and induce inflammatory responses in the infected cells (Sugiura et al., 2011; Zhao et al., 2011), and eventually an antiviral environment is set up to trigger an effective immune response (Wang et al., 2005; Johnson et al., 2006; Faul et al., 2010). Like most of the viruses, RABV has developed a strategy to counteract the antiviral effect of the type I IFN response (Chelbi-Alix et al., 2006; Rieder and Conzelmann, 2009). The N protein of RABV limits RIG-I signaling (Masatani et al., 2010), whereas the P protein of RABV inhibits IRF3 phosphorylation (Brzozka et al., 2005), suppresses STAT1 nuclear translocation (Vidy et al., 2005; Brzozka et al., 2006), and sequesters an antiviral protein, the promyelocytic leukemia (PML) protein in the cytoplasm (Blondel et al., 2010). Moreover, to a certain extent, the RABV can exploit the innate immune response to develop its immunoevasive strategy (Chopy et al., 2011b). Once RABV has entered the CNS, additional innate and adaptive immune mechanisms are required for attenuated viral clearance. These include the production of proinflammatory cytokines and chemokines by infected tissues (Phares et al., 2006), innate and CD4 T cell-mediated alterations in the blood-brain barrier (BBB) function that facilitate immune effector infiltration into the CNS, and production of antibodies in the CNS parenchyma (Roy et al., 2007; Hooper et al., 2011).

Post-exposure treatment of the disease with rabies vaccine is an effective way to prevent the spread of the virus from a peripheral site of exposure to the CNS. Infected individuals rarely develop the immune responses capable of clearing the RABV from nervous tissue, while a variety of attenuated rabies viruses can be readily cleared from the CNS tissues in animal models (Hooper et al., 2011). A wide variety of wild-type and laboratory-attenuated rabies viral strains are available for comparative antiviral immune system studies. Although certain mechanisms have already been implicated in the pathogenesis of the virulent RABV and clearance of attenuated RABV from the CNS (Wang et al., 2005; Phares et al., 2006; Roy et al., 2007; Faber et al., 2009; Hooper et al., 2009; Li et al., 2012; Gnanadurai et al., 2015), the differences in the host immune responses and pathogeny during infection by virulent and attenuated RABV strains still provide a vast scope to conduct precisely planned and more comprehensive investigations. Advances in the field of high-throughput RNA sequencing (RNA-Seq) technology has helped a lot in conducting the transcriptome-level studies to gain deep insights into the underlying mechanisms of gene regulations and networks (Yang et al., 2014). In this study, we found that the attenuated high-egg-passage Flury (HEP-Flury) strain can enter the brain through intranasal (i.n.) inoculation rather than intramuscular (i.m.) injection, and cause neurological symptoms followed by its clearance from CNS within 40 days after infection. Here, RNA-Seq has been used for the systematic analysis of global host transcriptional responses in the CNS of mice infected with attenuated HEP-Flury and virulent challenge virus standard-11 (CVS-11) strains. The differential regulations, of the genes and the signaling pathways that might contribute to the pathogenesis of the virulent rabies virus or the prevention of lethal encephalitis, were revealed.

## Materials and methods

### Cells, viruses, and animals

Mouse neuroblastoma (NA) cells were maintained in RMPI 1640 medium (Gibco, USA) which contains 10% fetal calf serum (FBS). CVS-11 and HEP-Flury were propagated in NA cells. Newcastle disease virus (NDV) vaccine strain LaSota was purchased from Winsun Pharm Company (Guangzhou, China). Female BALB/c mice were purchased from Center for Laboratory Animal Science, Southern Medical University (Guangzhou, China) and kept at the Laboratory Animal Center of South China Agricultural University.

### Animal infection and tissue collection

The mice were anesthetized with ketamine/xylazine (100/10 mg/kg, intraperitoneally) and given intranasal (i.n.) doses of 10^4^ focus-forming units (FFU) of either CVS-11 or HEP-FLury in 50 μL of phosphate buffered saline (PBS) and the mock-infected control mice were treated with PBS. Infected animals were observed twice per day for 50 days in order to find any rabies signs and symptoms. Body weight was also monitored and peripheral blood was obtained at different time intervals. Serum antibodies against rabies virus were measured using an ELISA (CitySynbiotics, USA), according to the manufacturer's instructions. Antibody titer expressed in EU/ml (equivalent units per ml) was calculated using the regression curve obtained from the WHO standard serum dilutions. At the indicated time points, mice were anesthetized and then perfused by intracardiac injection of PBS. The brain tissues were removed, lysed in TRizol® reagent (Invitrogen, USA) or flash frozen in liquid nitrogen before being stored at −80°C, for respective studies. All the procedures were conducted in accordance with the Public Health Service Policy on Humane Care and Use of Laboratory Animals, under protocols approved by the Animal Experimentation Ethics Committee (AEEC) of South China agricultural University (Permit Numbers: 2015B004 and 2015B031).

### Whole-transcriptome analysis of mouse brain RNA

Total RNAs, isolated from the mouse brains (*n* = 3) representing the groups of CVS-11 infected mice at 10 days post-infection (dpi), HEP-Flury infected mice at 10, 18, 25 dpi and mock-infected mice, were used for transcriptome analysis. Total RNA was extracted using the RNeasy Midi kit (Qiagen, Germany). RNA-Seq was performed by Novogene Bioinformatics Technology Co., Ltd (Beijing, China) following standard conditions. Sequencing libraries were generated and processed for high-throughput sequencing on an Illumina Hiseq 2000 platform and 100 bp single-end reads were generated according to the standard Illumina protocols. Raw data (raw reads) of fastq format were first processed through in-house perl scripts. In this step, clean data (clean reads) were obtained by removing reads containing adapter, reads containing ploy-N and low quality reads from raw data. All the downstream analyses were based on the clean data with high quality. Single-end clean reads were aligned to the mouse reference genome using TopHat v2.0.9. HTSeq v0.5.4p3 software was used to count the number of reads mapped against the reference sequence of each gene. The number of Reads Per Kilobase of exon model per Million mapped reads (RPKM) is considered as the effective sequencing depth (Mortazavi et al., 2008). The RPKM of each gene was calculated based on the length of the gene and reads count mapped to that particular gene.

Differential expression analyses of the conditions in each group were performed using the R package DESeq (1.12.0) (Anders and Huber, 2010). The DESeq package provides statistical routines for determining differential expression in digital gene expression data, using a model based on the negative binomial distribution. The resulting *P*-values were adjusted using the Benjamini and Hochberg's approach for controlling the False Discovery Rate (FDR). The genes with an adjusted *P* < 0.05 and absolute fold change greater than 2.0 were considered as differentially expressed. Venn diagrams were created using VENNY 2.0.2 (http://bioinfogp.cnb.csic.es/tools/venny/index.html). All sequencing data were deposited in NCBI's Sequence Read Archive (SRA) database (accession number: SRP061600).

### Gene ontology (GO) and pathway analysis

GO enrichment and Kyoto Encyclopedia of Genes and Genomes (KEGG) pathway analysis was performed on the sets of differentially regulated genes using the Database for Annotation, Visualization, and Integrated Discovery (DAVID) version 6.7 (Huang da et al., 2009). The resulting clustering was then limited to an enrichment score of >1.00 and a *P* < 0.05. The FDR for multiple testing was performed by the Benjamini and Hochberg method invoked within DAVID.

### Quantitative real-time RT-PCR (RT-qPCR)

Three mice per group were euthanized at each indicated time point. The cDNA was prepared from purified RNA using ReverTra Ace qPCR RT master mix with a genomic DNA (gDNA) remover kit (Toyobo, Japan). The RT-qPCR assay was carried out using THUNDERBIRD SYBR qPCR Mix (Toyobo, Japan) according to the manufacturer's instructions using specific primers for the targets. The Primer sequences used for the amplification of genes are listed in Supplementary Table 1. The mRNA copy numbers of a particular gene were normalized to the housekeeping gene glyceraldehyde-3-phosphate dehydrogenase (GAPDH) using the 2^−Δ*ΔCT*^ method (Livak and Schmittgen, 2001).

### Western blotting

The mouse brain tissues were lysed in the lysis buffer (50 mM Tris-HCl, pH 7.4, 150 mM NaCl, 1 mM EDTA, 1% NP-40) supplemented with 0.02 mM phenylmethanesulfonyl fluoride (PMSF). After electrophoresis, the separated proteins were then transferred onto a polyvinylidene difluoride (PVDF) membrane. Rig-I monoclonal antibody (Cell Signaling Technology, USA), STAT1 and p-STAT1 polyclonal antibodies (Bioworld Technology, USA) and horseradish peroxidase (HRP)-conjugated goat anti-mouse or rabbit IgG (Sigma, USA) were used, respectively, as the primary and secondary antibodies at the dilutions recommended by each manufacturer. The protein bands were detected by BeyoECL plus (Beyotime Biotech, China).

### Statistical analysis

The significance of differences between groups was evaluated using one-way analysis of variance (ANOVA) and Tukey's multiple-comparison test. The graphs were plotted and analyzed using Origin 8.0 (OriginLab Corporation, USA).

## Results

### Different outcomes of CVS-11 and HEP-flury infections

**Figure 1:**
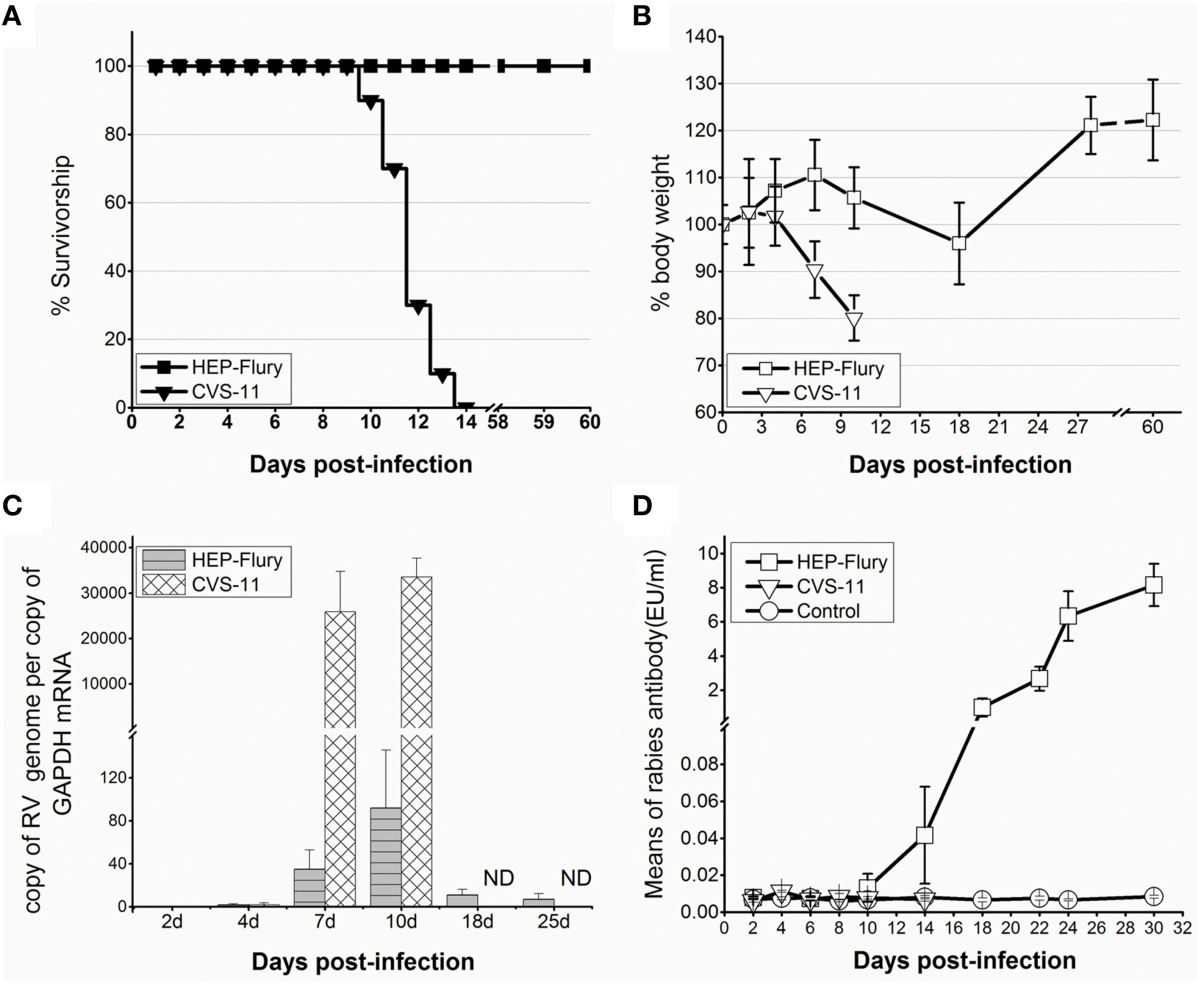
**A comparison of the outcomes of pathogenic CVS-11 and attenuated HEP-Flury infections**. The groups of BALB/c mice (*n* = 10) were infected with 10^4^ FFU of either CVS-11 or HEP-Flury i.n. and PBS mock-infected mice served as the controls. The mice were observed for 50 days, the survival **(A)** and the body weight **(B)** are presented. Three mice per group were euthanized at each indicated time point, and the quantity of genomic RNA in brain tissue samples was measured by RT-qPCR **(C)**. The anti-rabies antibody (RV-Ab) titers in serum were determined as described in Materials and Methods, and are presented as mean titers **(D)**. ND, not detected.

According to the previous reports and our experimental evidences, attenuated rabies strain HEP-Flury cannot invade into the brain to cause rabies via i.m. injection (Takayama-Ito et al., 2006). In this study, the i.n. route was chosen because it is the most efficient method of rapidly promoting RABV entry into the brain while avoiding the injury that induces innate immune mechanisms that accompany intracranial (i.c.) administration (Li et al., 2012). The mice infected with CVS-11 developed typical neurologic signs of rabies, including ataxia and paralysis, and all died between 10 and 14 days post-infection (dpi) (Figure 1A). On the other hand, HEP-Flury infected mice showed subtle clinical signs and loss of body weight (about 15%) between 6 and 24 dpi, but completely recovered in 27 days after infection (Figure 1B). As shown in Figure 1C, the viral genomic RNA was readily detected in the brain tissue of HEP-Flury and CVS-11 infected mice at 4 dpi. The genomic RNA levels of both groups increased between 4 and 10 dpi, but remained lower and declined significantly at 18 dpi in the brain tissue of the HEP-Flury infected mice. Comparison of the anti-rabies antibody (RV-Ab) titers at different days after infection revealed that HEP-Flury infected mice developed RABV-specific immunity at 10 dpi (Figure 1D), while even low levels of RV-Ab were undetectable in CVS-11 mice after infection. The HEP-Flury infected mice produced high RV-Ab titers (>0.6 EU, the threshold level for protection against rabies) at 18 days after challenge (Figure 1D). The serum RV-Ab levels continued to increase in the HEP-Flury infected animals, reaching the level of 8 EU at 30 dpi. These results revealed that the CVS-11 and HEP-Flury were able to invade the brain to cause neurological symptoms by the i.n. route. The CVS-11 infected mice showed more severe clinical symptoms and higher CVS-11 replication rate, and eventually died. In the meanwhile, the HEP-Flury viral strains were able to activate host adaptive immunity better than the CVS-11 viral strains, and could be eliminated from the CNS.

### Global transcriptome analysis revealed the immune regulation of CNS in rabies pathogenesis and protection

**Figure 2:**
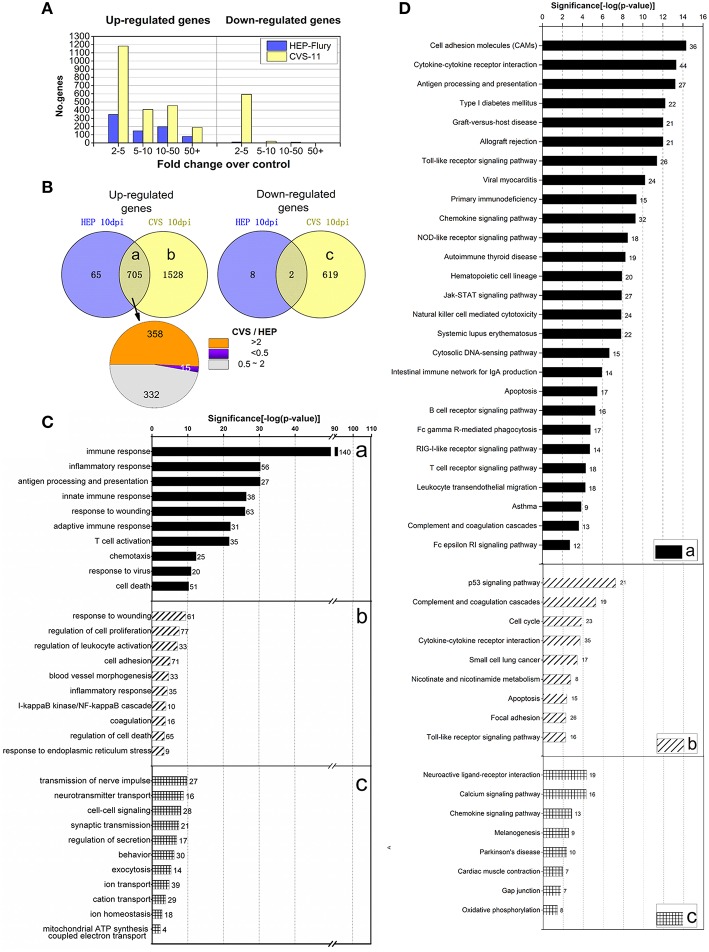
**Differential gene expression profiles in the mouse brain tissue following CVS-11 and HEP-Flury infection at 10 dpi**. **(A)** Graph showing the number and fold change of up-regulated and down-regulated genes (a threshold of ≥2-fold change in expression levels and adjusted *P* < 0.05) following virus infection. **(B)** A Venn diagram depicting the overlap of differentially expressed genes, and pie chart showing the number of genes that were up-regulated at different levels. **(C)** The representative GO terms of the common or unique, differentially regulated genes. **(D)** The representative KEGG pathways of the common or unique, differentially regulated genes. For more details, see the Supplementary Tables 2–9.

**Figure 3:**
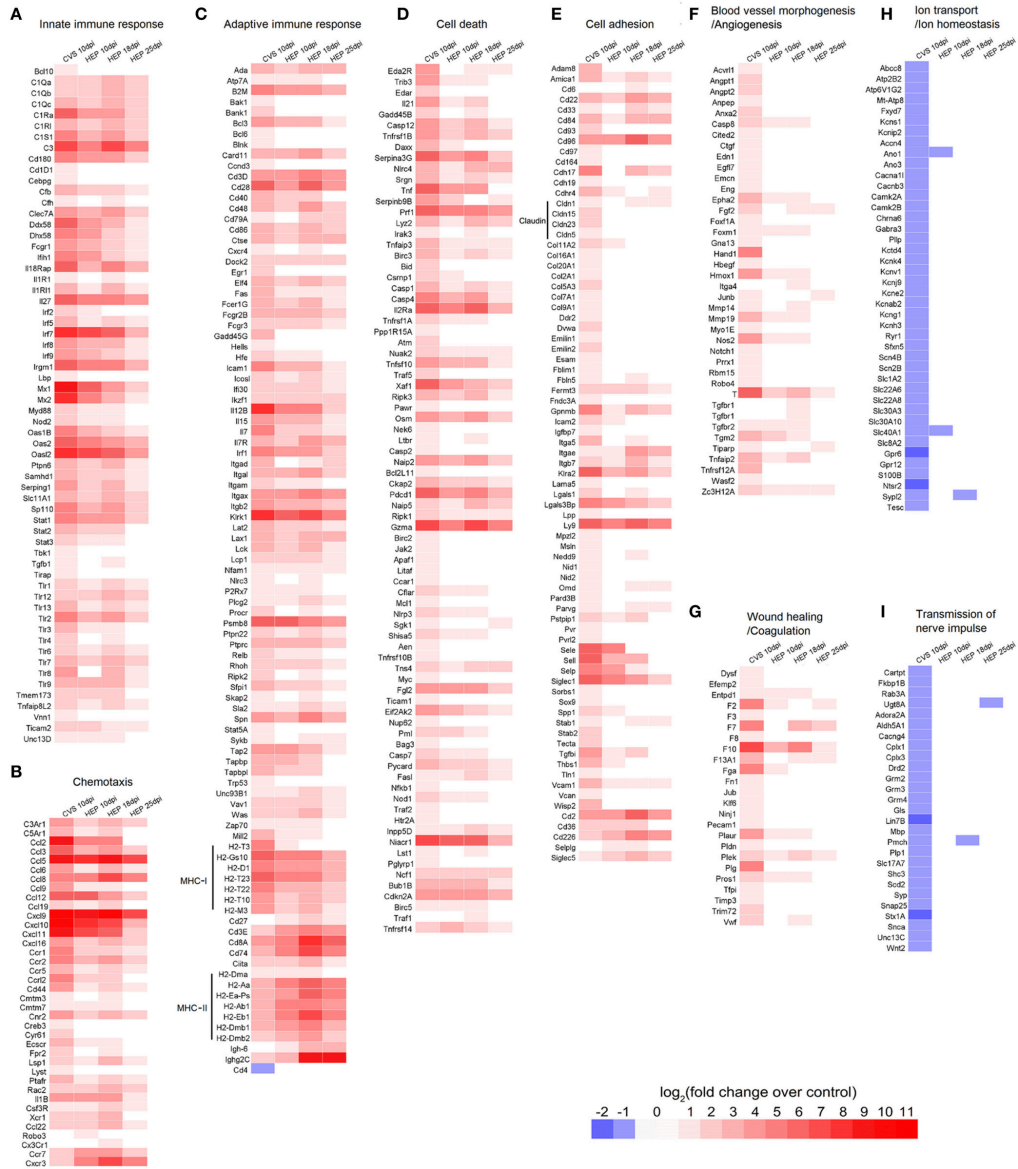
**The representative heat maps showing changes in expression of genes following CVS-11 or HEP-Flury infection. (A)** Innate immune response. **(B)** Chemotaxis. **(C)** Adaptive immune response. **(D)** Cell death. **(E)** Cell adhesion. **(F)** Blood vessel morphogenesis or angiogenesis. **(G)** Wound healing or coagulation. **(H)** Ion transport or homeostasis. **(I)** Transmission of nerve impulse. For more details, see the Supplementary Tables 2–6.

To obtain the comprehensive insight into the host response mechanisms in the CNS that may influence the outcome of the infection, we performed comparative whole transcriptome analysis of the RABV (CVS-11 and HEP-Flury) infected mouse brain tissues that were collected at 10 days after the infection. A total of 2233 genes were found to be up-regulated and 621 genes were down-regulated (a threshold of ≥2-fold change in expression levels and adjusted P ≤ 0.05) in the brain tissue following infection with CVS-11 at 10 days after infection, and 770 genes were up-regulated, 10 gene were down-regulated following infection with HEP-Flury (Figures 2A,B; also, see details in the Supplementary Tables 2, 3). The magnitude of the altered genes that were down-regulated was much lower than up-regulated genes (Figure 2A). Among the 705 commonly up-regulated genes, 358 genes were at least 2-fold up-regulated in the CVS-11 infected mice compared with the HEP-Flury infected mice, and are thought to be involved in the innate immune and inflammatory responses (Figures 2B,C, 3A,B). Another group of 15 genes, mainly involved in antigen processing and presentation of peptide antigens via MHC class II, were at least 2-fold up-regulated in the HEP-Flury infected mice compared with the mice infected with CVS-11 (Figures 2B, 3C). In addition, many genes showed significant dysregulation only during CVS-11 infection, including 1528 up-regulated genes involved in regulation of cell proliferation, response to wounding, cell adhesion, blood vessel morphogenesis, angiogenesis and coagulation (Figures 2C,D, 3D–G). Some of these genes might be involved in the pathological reactions of rabies. In CVS-11 infected mouse brain tissue, 619 genes with roles in synaptic transmission and ion transport, mapped to the neuroactive ligand-receptor interaction, calcium signaling pathway and Parkinson's disease, were found to be exclusively down-regulated (Figures 2C,D, 3H,I). Sixty-five genes uniquely up-regulated in the brain of HEP-Flury infected mice are mainly involved in regulation of lymphocyte activation, including genes encode CD5 antigen, immunoglobulin heavy chain 6, linker for activation of T cells (LAT), Interleukin (IL)-27 receptor alpha and chemokine (C-X-C motif) receptor 3 (CXCR3) (Supplementary Table 6). More details of Gene Ontology (GO) enrichment and Kyoto Encyclopedia of Genes and Genomes (KEGG) pathway analysis can be found in Supplementary Tables 7–9.

**Figure 4:**
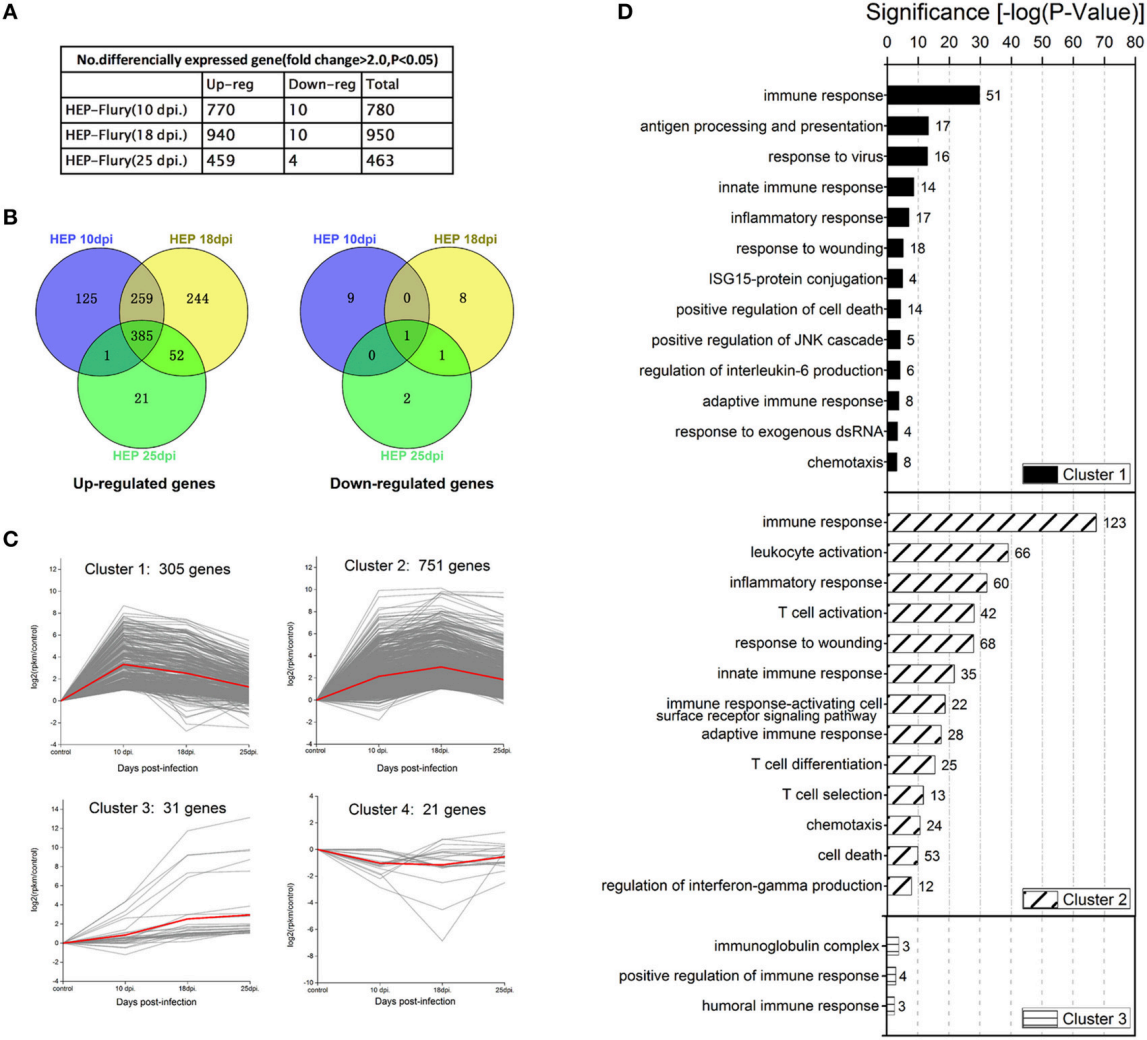
**Differential gene expression profiles in the mouse brain tissue following HEP-Flury infection at 10 dpi, 18 dpi and 25 dpi. (A)** The number of differentially expressed genes in HEP-Flury infected mice at 10, 18, and 25 dpi. **(B)** Venn diagrams showing the overlap of differentially expressed genes in the infected mouse brains. **(C)** Graphs showing 4 distinct clusters of the differentially expressed genes. **(D**) The representative GO terms of the altered genes in the 4 distinct clusters. For more details, see the Supplementary Tables 3–5, 10–12.

In order to further understand the host mechanisms involved in the prevention of the lethal outcomes, and to obtain comprehensive information on the expression kinetics of the particular host genes in the brain during clearance of HEP-Flury, comparative transcriptome profiling of the HEP-Flury infected mouse brain was performed at day 18, when the most obvious neurological symptoms were observed, and at day 25, after complete recovery. Overall, most of the genes with abnormal expression were up-regulated, only a few genes were down-regulated (Figures 4A,B). The largest amount of up-regulated genes emerged at 18 dpi, and might be associated with the obvious clinical symptoms. According to the temporal expression pattern at 10, 18, 25 dpi, we subdivided altered genes into 4 distinct clusters (Figure 4C). Cluster 1, consisted of the genes that showed the highest level at 10 dpi, and contained 305 genes involved in different cellular processes including antigen processing and presentation, innate immune response and inflammatory response (Figure 4D). Cluster 2, in which gene expression was increased until 18 dpi, had 751 members, including genes implicated in leukocyte activation and inflammatory response (Figure 4D). Cluster 3 consisted of 31 genes that were up-regulated consistently from 10 to 25 dpi, including 4 immunoglobulin genes. Cluster 4, consisted of 21 genes that were expressed at the highest levels in mock-infected mice, including genes encode hemoglobin beta chain, gastrokine 3 and gap junction gamma-2 protein.

### HEP-flury infection induced the early expression of genes associated with RIG-I-like receptor signaling pathway and activated the adaptive immunity completely

**Figure 5:**
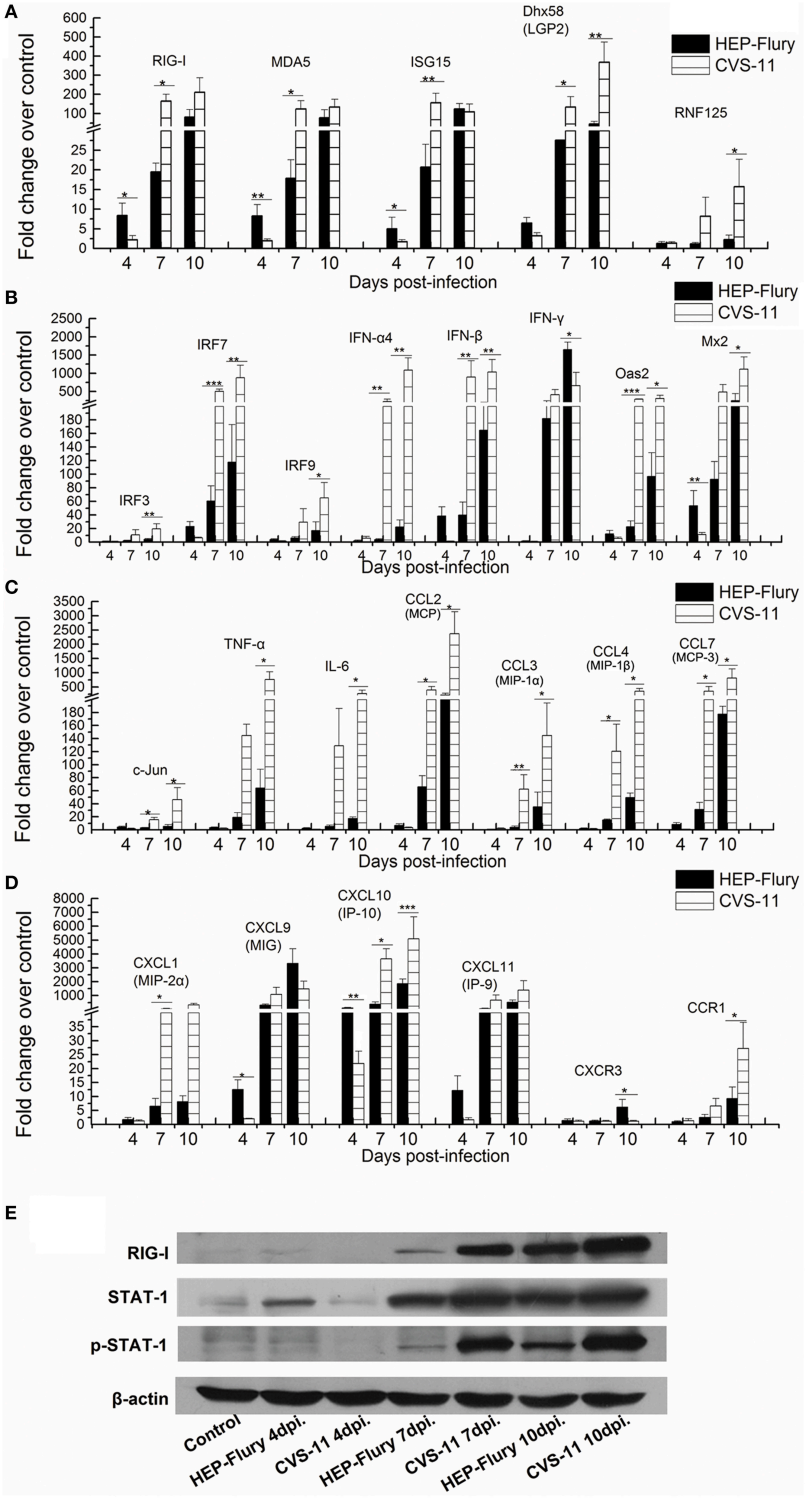
**RT-qPCR and western blot analyses of the temporal alterations of genes associated with RIG-I-like receptor signaling pathway**. RT-qPCR analyses of the genes involved in the innate immunity **(A,B)** or chemotaxis **(C,D)**, and western blot detection of RIG-I, STAT, and p-STAT **(E)**. The asterisks indicate significant differences in the copy numbers between different RABV infections at same time points determined by one-way ANOVA and Tukey's multiple-comparison test. ^***^,^**^, and ^*^ represent *P*-values of less than 0.001, 0.01, and 0.05, respectively.

In order to analyze the dynamic expression of important immune-related genes, we used RT-qPCR to compare the expression levels of several innate and adaptive immune effectors in the mouse brain tissues following CVS-11 or HEP-Flury challenge at 4, 7, and 10 dpi. The RT-qPCR analysis revealed that the RIG-I, MDA5 and their regulators such as RING finger protein 125 (RNF125), Interferon-stimulated gene 15 (ISG15), DExH (Asp-Glu-X-His) box polypeptide 58 (Dhx58, also known as LGP2) were up-regulated following CVS-11 and HEP-Flury infections (Figure 5A). Most of these genes were up-regulated more rapidly and strongly in the HEP-Flury infection than the CVS-11 at 4 dpi besides RNF125. Whereas, all of these genes were largely expressed in both groups after 7 dpi and overexpressed more intensively following CVS-11 infection. Interferon (IFN) genes IFN-α4, IFN-β, IFN regulatory factors (IRF-3, IRF-7, and IRF-9), and IFN-induced genes were all significantly up-regulated in CVS-11 or HEP-Flury infected mouse brains after 7 dpi, and more intensively in CVS-11 infected mouse brains (Figure 5B). Notably, the IFN-β and IRF-7 were up-regulated earlier at day 4 after HEP-Flury infection but not in CVS-11 infection, and IFN-γ was significantly elevated in the HEP-Flury infected mouse brains than the CVS-11 infected mouse brains after 10 dpi (Figure 5B). The proinflammatory chemokines in both the families C-C and C-X-C, including CCL2 (MCP), CCL3 (MIP-1α), CCL4 (MIP-1β), CCL7 (MCP-3), CXCL1 (MIP-2α), CXCL9 (MIG), CXCL10 (IP-10), and CXCL11 (IP-9) were all up-regulated with both CVS-11 and HEP-Flury infections and reached peak levels at 10 dpi, some even increased more than 1000-fold (Figures 5C,D). Among them, CXCL9, CXCL10, and CXCL11 were elevated more significantly following HEP-Flury infection at 4 dpi than CVS-11 infection. The CXCR3, a receptor of CXCL9, CXCL10, and CXCL 11, was elevated only in the brains of the HEP-Flury infected mice at 10 dpi (Figure 5D). The mRNA levels specific for c-Jun, tumor necrosis factor alpha (TNF-α) and interleukin-6 (IL-6) were increased more significantly in the CVS-11 infected mouse brains than the HEP-Flury infected mouse brains after 7 dpi (Figure 5C). Furthermore, the western blot analysis with antibodies against RIG-I, STAT-1, and p-STAT-1 showed that they were all gradually up-regulated in response to infection with both viruses, indicating that either CVS-11 or HEP-Flury can engage RIG-I and IFN-α/β during the infection, resulting in the innate immune signaling (Figure 5E). A intense band of STAT-1 and a weak band of RIG-I appeared earlier at day 4 after the HEP-Flury infection, which indicated that the RIG-I-like receptor signaling pathway was activated quickly by HEP-Flury infection, this result was consistent with the results of RT-qPCR.

**Figure 6:**
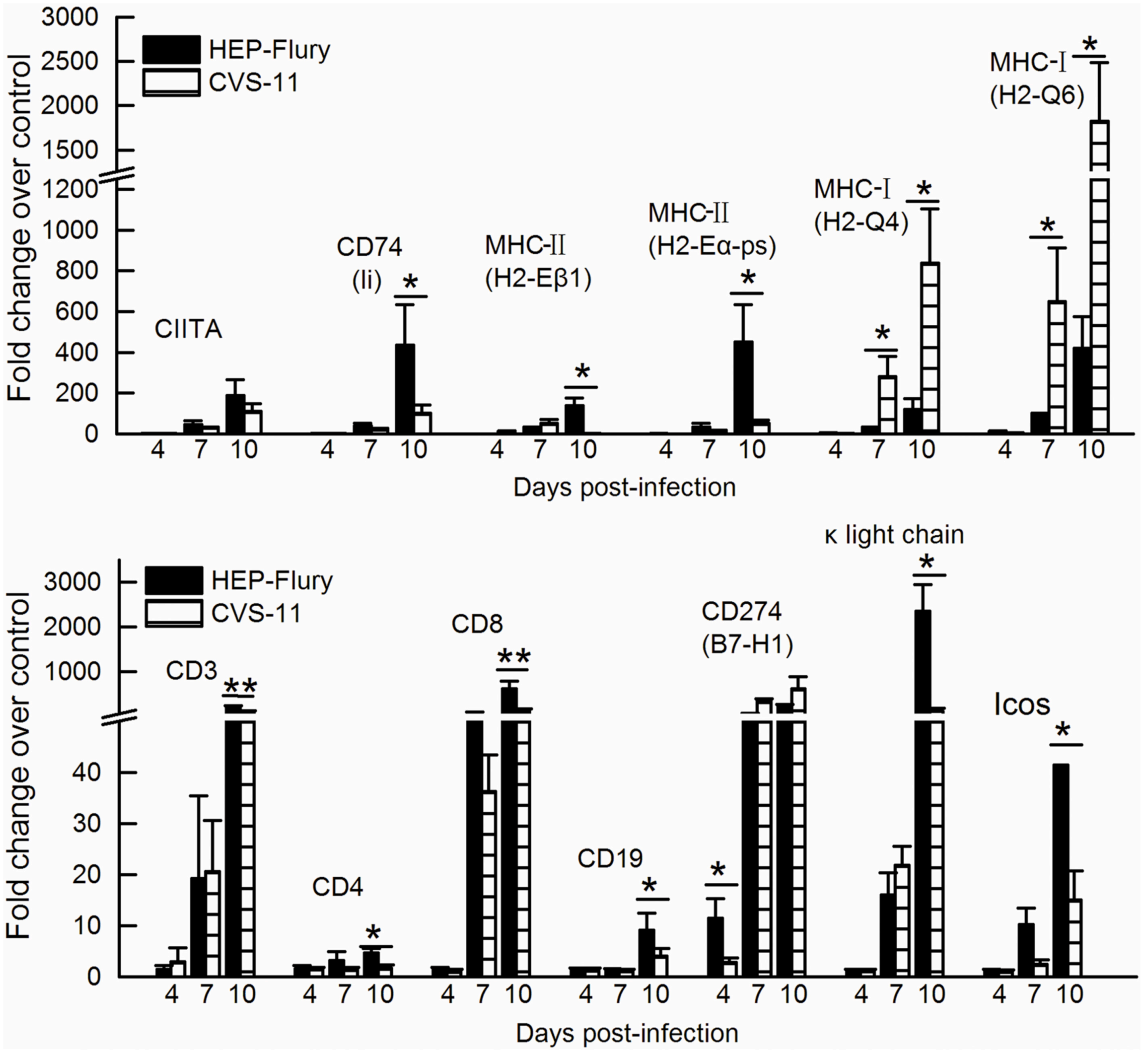
**RT-qPCR analyses of the temporal alterations of genes involved in the adaptive immunity in the mouse brains with CVS-11 or HEP-Flury infections**. The asterisks indicate significant differences in the copy numbers between different RABV infections at same time points determined by one-way ANOVA and Tukey's multiple-comparison test. ^**^ and ^*^ represent *P*-values of less than 0.01 and 0.05, respectively.

Corresponding to the above results that IFN-γ was elevated at higher levels in the HEP-Flury infected mouse brains, the IFN-γ-induced MHC class II transactivator gene (CIITA), MHC class II associated invariant chain (Ii; CD74) and MHC class II were all elevated more significantly following the HEP-Flury infection at 10 dpi than the CVS-11 infection (Figure 6). On the other hand, expression levels of the MHC class I were higher following CVS-11 infection at either 7 or 10 dpi. The mRNAs specific for lymphocyte surface markers, including CD19, CD3, CD4, CD8, were up-regulated more significantly in response to the infection with HEP-Flury at 10 dpi (Figure 6). Icos, which is essential for the effective T-helper-cell responses, was more significantly increased after the HEP-Flury infection at 10 dpi. The κ light-chain mRNA, indicative of antibody production, was elevated to higher levels in the HEP-Flury infected mice at 10 dpi.

### The innate immunity pre-activated by HEP-flury or NDV infection can effectively prevent the CVS-11 from invading in the CNS

**Figure 7:**
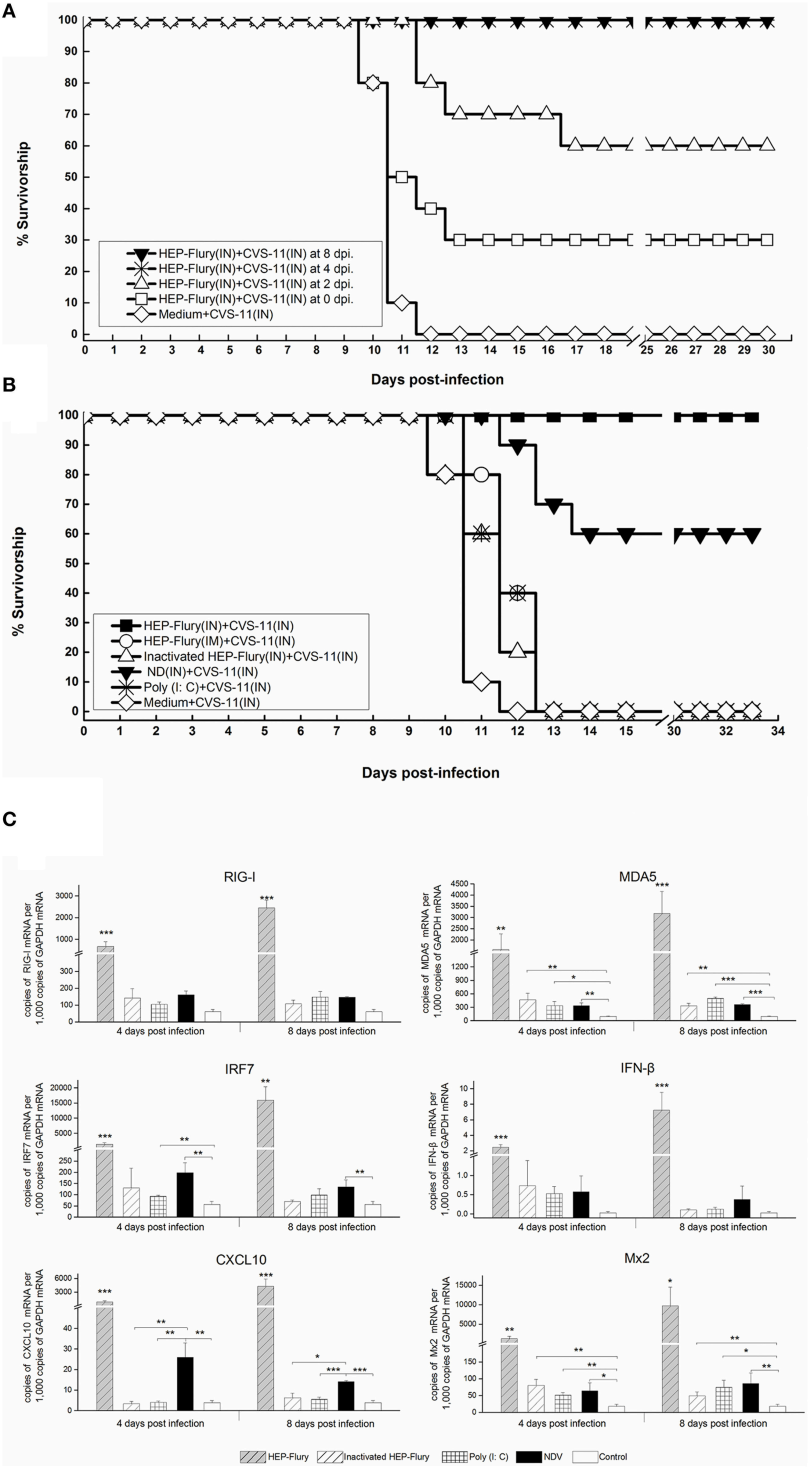
**Intranasal inoculation with the HEP-Flury or NDV, promotes survival in CVS-11 challenged mice. (A)** The groups of 10 adult mice infected i.n. with 10^4^ FFU of HEP-Flury in advance and then infected with 10^4^ FFU of CVS-11 i.n. at 0, 2, 4, 8 days post HEP-Flury infection. **(B)** The groups of 10 adult mice pretreated respectively with 10^4^ FFU of HEP-Flury (i.n. inoculation), HEP-Flury (i.m. injection), β-propiolactone-inactivated HEP-Flury (i.n. inoculation), 10^7^ EID_50_ NDV vaccine strain LaSota (i.n. inoculation) and 100 μg poly (I:C) (i.n. inoculation, once a day), and then infected with 10^4^ FFU of CVS-11 i.n. at 4 days after pretreatment. The mice were observed for 50 days. **(C)** The RT-qPCR analysis of the alterations of several innate immunity genes in the mouse brain tissues of different groups. ^***^,^**^, and ^*^ represent *P*-values of less than 0.001, 0.01, and 0.05, respectively.

The above results proved that the HEP-Flury infection can activate the innate immunity earlier and trigger the adaptive immune responses more completely as compared to the CVS-11 infection. To further investigate whether the intermodulation or crosstalk existed in the immune responses following CVS-11 and HEP-Flury infections, we infected mice i.n. with 10^4^ FFU of HEP-Flury in advance and then challenged the mice with lethal doses of CVS-11 i.n. at 0, 2, 4, 8 days after the HEP-Flury infection. Unexpectedly, the mice challenged by CVS-11 at 4 and 8 days post HEP-Flury infection showed 100% survival (Figure 7A). Those challenged at 0 and 2 days post HEP-Flury infection achieved 30 and 60% survival, respectively. Only HEP-Flury was detected in all brains of survived mice. A parallel group of CVS-11 infected mice at 2 days after HEP-Flury infection was set up and brains were removed at 8 days post CVS-11 infection. Detection with the specific primers for HEP-Flury or CVS-11 showed 100% HEP-Flury-positive and 50% CVS-11-positive, indicating that once CVS-11 enters the brain, mice will eventually die even if infected with HEP-Flury in advance. Based on our findings, we speculate that the HEP-Flury infection may initiate some immune responses to prevent CVS-11 entry into the brain but not clearance from CNS. In order to distinguish which immune responses prevent the development of rabies, the mice were infected i.n. with HEP-Flury and NDV vaccine strain, treated i.n. with inactivated HEP-Flury and Poly (I: C), or injected i.m. with HEP-Flury at 4 days before challenge with CVS-11, all mice that survived belonged to the HEP-Flury and NDV pre-infected groups but not others (Figure 7B). The survival rate of the NDV infected group was 60%, indicating that the innate immunity plays a critical role in preventing CVS-11 from invading or progressing in the CNS. RT-qPCR was used to compare the expression of several innate immune effectors, that were up-regulated earlier during HEP-Flury infection in the previous experiments, in the mouse brain tissues of different groups (Figure 7C). The results revealed that only CXCL10 was specifically and significantly elevated in the HEP-Flury or NDV pre-infected mice (Figure 7C), suggesting that CXCL10 may play a role in blockage of early CVS-11 infection of the CNS. More extensive studies will be required to identify the critical innate immune factors for protection against the CNS invasion of RABV.

## Discussion

The clinical outcome of viral infections depends on the balance between viral replication and the host immune response. The uncontrolled replication of virus in the CNS leads to the disease and ultimately death. In this study, during the process of infection, the attenuated HEP-Flury replicated more slowly, triggered the innate immunity earlier and achieved the adaptive immunity more completely than the virulent CVS-11 strain, leading to the subtle clinically observable signs and clearance from the CNS. Our results showed that the type I IFN response is continuously triggered in either CVS-11 or HEP-Flury infected mice, although RABV dampens type I IFN signaling by inhibiting IRF3 phosphorylation and STAT1 translocation (Brzozka et al., 2005, 2006; Vidy et al., 2005). The RABV viral signature RNA, such as the 5′-triphosphate RNAs, are detected by RIG-I protein, leading to RIG-I activation and interaction with the mitochondrial protein IFN-β promoter stimulator-1 (IPS-1) (Faul et al., 2010). Indeed, many genes that mapped to the RIG-I-like receptor signaling pathway were remarkably up-regulated in both CVS-11 and HEP-Flury infections (Figure 2D). The only differences were that RIG-I, MDA5, ISG15 were elevated earlier in the HEP-Flury infected mouse brains, whereas LGP2 and RNF125, two negative regulators of RIG-I/MDA5 signaling were increased at higher levels in the CVS-11 infected mouse brains at a later stage (Figure 5A). Regardless of the differences, a large number of IFN effector genes, IFN stimulated genes, inflammatory cytokines and chemokines were generally up-regulated in both infections. The 5′-triphosphate viral dsRNA and leader RNAs of RABV are the main pathogen-associated molecular patterns recognized by RIG-I/MDA-5 (Faul et al., 2010; Jensen and Thomsen, 2012). Yang et al. have found that a reduced level of *de novo*-synthesized leader RNA was generated in dendritic cells infected with the wild-type RABV compared to the laboratory-attenuated RABV (Yang et al., 2015). Therefore, we speculate that the level of *de novo*-synthesized leader RNA generated in the CNS infected by CVS-11 may be lower than in the HEP-Flury-infected CNS at the early stage, and it would be gradually elevated along with the increase in the viral load. In addition to the RIG-I-like receptor signaling pathway, HEP-Flury and especially CVS-11 can also stimulate the expression of genes encoding the TLRs, such as TLR-1, TLR-2, TLR-3, TLR-4, TLR-6, TLR-7, TLR-9, TLR-12, and TLR-13. It has been reported previously that the viral surface glycoproteins can activate TLRs (Li et al., 2011), suggesting the involvement of some other components of the RABV or stress proteins in the activation of TLR pathways and IFN-regulatory factors besides the viral genomic RNA. Previous studies have shown that the animals with RABV infections, pretreated or simultaneously treated with IFN or IFN inducers, could control RABV infection (Postic and Fenje, 1971; Marcovistz et al., 1987). In our present study, intranasal inoculation with the HEP-Flury or NDV promotes survival in CVS-11 challenged mice. Similar to RABV, NDV is also a non-segmented negative-stranded RNA virus and generates 5′-triphosphate viral RNA, which can be recognized by RIG-I/MDA-5 (Hornung et al., 2006; Sun et al., 2013; Oh et al., 2016), and efficiently triggers the production of IFN. Our results indicate that the innate immunity activated by certain RNA virus infections can effectively prevent virulent RABV from spreading to the CNS causing fatal encephalitis before the adaptive immunity is induced. Although the mechanisms and conditions for the innate immune protection need further investigations, additional treatment with suitable innate immunity activators might play a positive role in prophylaxis of rabies after exposure, especially in the higher-risk exposures like bites on the face.

Many inflammatory cytokines and chemokines were highly up-regulated in the HEP-Flury or (particularly) in the CVS-11 infected mice. It is possible that the interactions among neurons, astrocytes, microglia, and infiltrating T cells are responsible for the up-regulation of so many chemokines in RABV infections (Wang et al., 2005). Although the chemokines have direct antiviral activities in the recruitment of inflammatory cells to the site of infection to kill the virus or virus-infected cells (Melchjorsen et al., 2003), overexpression of CCL5 (RANTES) or CXCL10 (IP-10) increased RABV pathogenicity by causing neurological diseases, which is due to the excessive infiltration and accumulation of the inflammatory cells in the CNS (Zhao et al., 2009). However, recombinant RABVs expressing CCL3, CCL22 (MDC), as well as GM-CSF can further attenuate RABV (Zhao et al., 2010; Niu et al., 2011). Our results showed that the majority of chemokines and receptors were up-regulated more strongly in the CVS-11 infection than the HEP-Flury at a later stage, besides CCL22, CXCR3, CXCR6, and CCR7 (Figure 3, Supplementary Tables 2, 3). On the other hand, the expression of CXCL10 was induced earlier following HEP-Flury or NDV infection, suggesting its positive role in protection against initial infection of RABV. In West Nile virus (WNV) infection, it is neuronal CXCL10 that directs CD8^+^ T Cell recruitment and control of WNV encephalitis (Klein et al., 2005). Therefore, the roles of chemokines in rabies pathogenesis and protection deserve further study.

It has been reported that the infection with wild-type (wt) SHBRV does not induce MHC class II expression in the neurovasculature (Roy et al., 2007; Hooper et al., 2011). A recent study showed that the MHC class II was not up-regulated in dendritic cells (DCs) after infection with the wt RABV *in vitro*, which demonstrates that the wt RABV evades DC-mediated immune activation (Yang et al., 2015). In this study, MHC class II positive regulators IFN-γ, transactivator CIITA and MHC class II-associated invariant chain (CD74), which is a type II integral membrane protein essential for proper MHC class II folding in the endoplasmic reticulum (Matza et al., 2003), were suppressed to some extent at 10 days after the CVS-11 infection compared with the HEP-Flury infection. In addition, the MHC class II negative regulators including TGF-β and IFN-β (Ting and Trowsdale, 2002) were elevated at higher levels following CVS-11 infection at 10 dpi. In accordance with these observations, the expression of MHC class II was impaired in the mouse brain tissues infected with CVS-11, while their expression was induced in the HEP-Flury infected brain tissues. The presentation of the peptides to T cells by MHC class II molecules is of critical importance in specific recognition to a pathogen by the immune system and the expression levels of MHC class II directly influence the T lymphocyte activation (Li et al., 2009). In addition, the less elevated mRNA of immunoglobulin κ light chain and failure to produce the protective antibodies in CVS-11 infected mice suggest that the virulent RABV may impair the MHC class II antigen presentation and activation of T cells to inhibit the adaptive immune responses. It has been reported that some viruses have evolved elaborate strategies to disrupt MHC class II-mediated T cell responses by inhibiting MHC class II expression (Moutaftsi et al., 2002; Li et al., 2009). Our findings provide a clue to elucidate a strategy to inhibit the adaptive immunity by the virulent RABVs.

**Figure 8:**
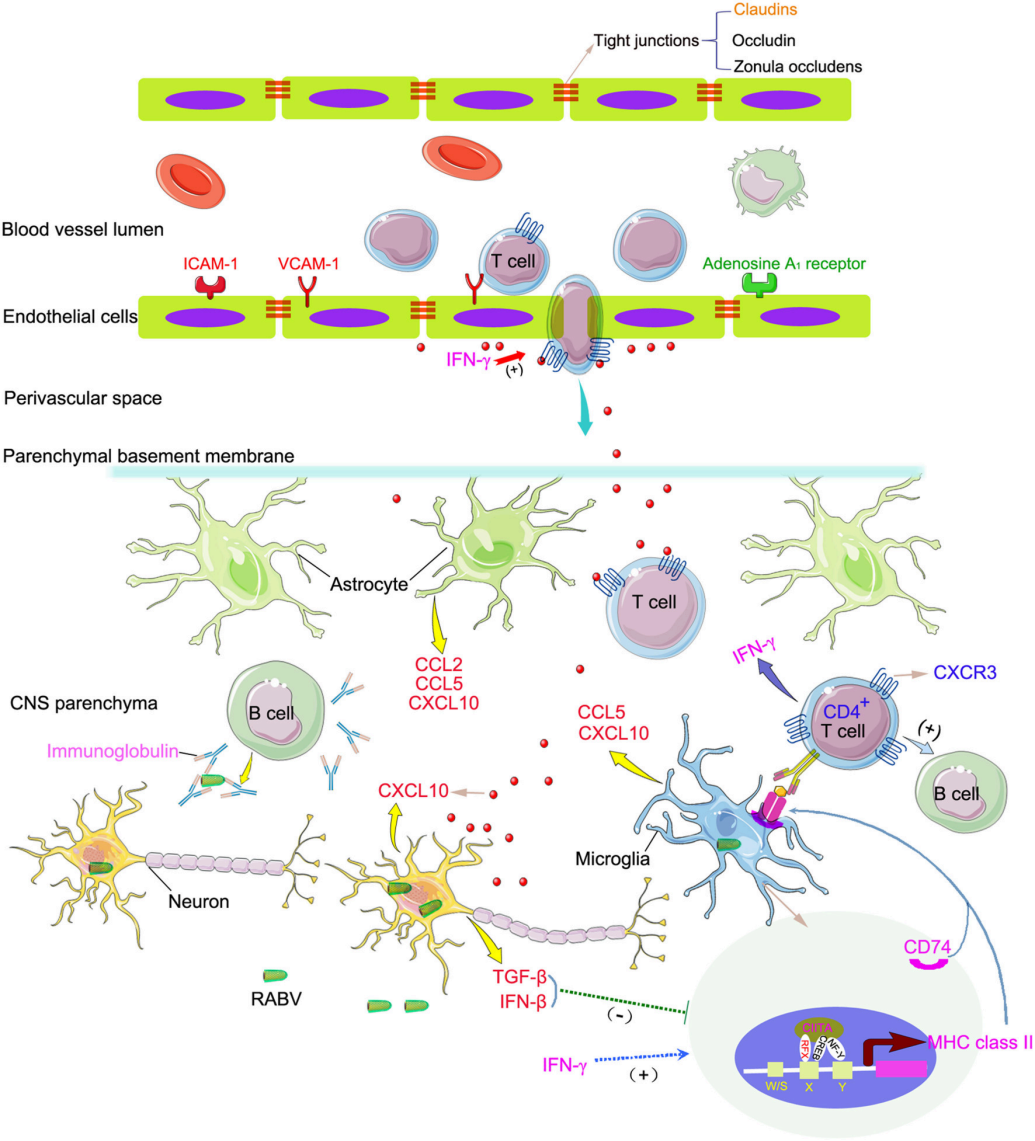
**Schematic overview of the differential expression of key genes involved in the immune responses to HEP-Flury and CVS-11 infection in the CNS**. Genes, commonly up-regulated in HEP-Flury and CVS-11 infected brains are marked in red, uniquely up- and down-regulated in CVS-11 infected brains are marked in orange and green, respectively. Genes, up-regulated more significantly in response to the infection with HEP-Flury are marked in pink, uniquely up-regulated in HEP-Flury infected brains are marked in blue. +, indicates activation. −, indicates inhibition. Figure was produced using the elements of Servier Medical Art (http://www.servier.com/Powerpoint-image-bank).

Histological studies have shown that the CNS of RABV infected mice was infiltrated by CD4^+^ and CD8^+^ T cells, with CD8^+^ T cells outnumbering CD4^+^ T cells from day 10 onwards (Galelli et al., 2000). Among the T cell population, the protection of RABV is conferred exclusively by CD4^+^ T cells because fatal encephalopathogeny develops only after the depletion of the CD4^+^ and not the CD8^+^ T cells (Galelli et al., 2000; Hooper et al., 2011). Noticeably, the CD4 mRNA was increased in the HEP-Flury infected mouse brains but was down-regulated in the brains of CVS-11 infected mice, and CD8 mRNA was up-regulated more significantly in response to the infection with HEP-Flury (Figures 3, 6). It has been reported that the pathogenic RABV strain associated with the silver-haired bat (SHBRV) fails to open the BBB and deliver immune effectors, including IFN-γ^+^ CD4 T cells, to CNS and leads to a lethal outcome (Roy et al., 2007). Furthermore, Chai et al found that the enhancement of BBB permeability in mice infected with attenuated RABV is associated with the reduction of tight junction (TJ) protein, such as claudin-5, occludin, and zonula occludens-1 (Chai et al., 2014). During the later stage of CVS-11 infection, the exclusively up-regulation of claudin-5, 15, and 23 (Figure 3) and down-regulation of adenosine A2a receptor, which has been indentified as a critical mediator of BBB permeability (Carman et al., 2011), implies an inability to enhance blood-brain barrier permeability and deliver immune effectors, especially the CD4^+^ T cells, to the CNS tissues in the CVS-11 infected mice. In addition, the immunosubversive molecules such as B7-H1 and HLA-G, which trigger death signaling in activated T cells expressing the corresponding ligands (Lafon, 2011), were up-regulated more significantly in CVS-11 infected mouse brains suggests that CVS-11 triggers the worse conditions for T cell survival. The chemokines CXCL9, CXCL10, and CXCL11 are structurally and functionally related IFN-γ-inducible proteins, each bind to the receptor CXCR3 expressed on activated CD4^+^ T cells and play a function in governing the migration of lymphocytes into the CNS (Muller et al., 2010). Previous studies have indicated that CXCR3 mediates CNS accumulation of antibody secreting cells during neurotropic coronavirus-induced encephalomyelitis (Marques et al., 2011). Here, CXCR3, up-regulated specifically in the mouse brain tissues infected with HEP-Flury, together with the higher expression level of INF-γ and MHC class II, help sketch out a hypothesis that cross talks between INF-γ, chemokines, MHC class II antigen presentation and CD4^+^ T cell signaling pathways play a pivotal role in the enhancement of BBB permeability and clearance of attenuated RABV from the CNS (Figure 8).

This is the first report that demonstrates the pathogenic RABV infection can induce the expression of genes, which encode extracellular matrix (ECM) proteins and play roles in blood vessel morphogenesis and angiogenesis. Angiogenesis and inflammation are frequently coupled in pathological situations such as arthritis, atherosclerosis and diabetes (Arroyo and Iruela-Arispe, 2010). The inflammatory response increases capillary permeability and induces endothelial activation, which, when persistent, results in capillary sprouting (Carmeliet, 2000; Arroyo and Iruela-Arispe, 2010). Inflammation-induced angiogenesis and the subsequent remodeling steps are largely mediated by ECM proteins and proteases (Arroyo and Iruela-Arispe, 2010). The delicate relationship between inflammation and blood vessel morphogenesis in the CNS following RABV infection deserves comprehensive investigations. Electrophysiological changes and dysfunction of ion channels have been observed in the human rabies patients (Iwata et al., 1999; Fu and Jackson, 2005; Mitrabhakdi et al., 2005). These alterations in neurophysiology might be due to the malfunction in neurotransmission. Many of the genes involved in synaptic transmission and ion transport are uniquely down-regulated in CVS-11 infection, some of them have been identified in previous proteomic analyses of RABV infection (Dhingra et al., 2007; Zandi et al., 2009). Alpha-2 adrenoreceptor was significantly decreased in RABV infection, which could possibly contribute to clinical features of rabies, including hyperexcitability and aggressive behavior (Iwata et al., 2000). 5-Hydroxytryptamine receptor (5-HT) was also found to be significantly decreased in the later stages of RABV infection (Akaike et al., 1995). In addition to adrenoceptor and 5-HT, adenosine A2a receptor, alpha-adrenergic receptor, oxytocin receptor, dopamine receptor 2, glutamate receptor and ryanodine receptor 1 were found to be down-regulated in CVS-11 infection. These alterations might lead to the degenerative changes and dysfunction of neuronal processes. On the other hand, two neural activity related genes, neuronal PAS domain protein 4 (Npas4) and activity regulated cytoskeletal (Arc)-associated protein, were progressively up-regulated in HEP-Flury infected mouse brains and sustained to 25 dpi. The expression of Npas4 was inhibited in the CVS-11 infected brains. It is a transcription factor, highly expressed in the brain, and regulates the formation and maintenance of the inhibitory synapses as well as excitatory-inhibitory balance within the neural circuits (Spiegel et al., 2014). The Arc is required for homeostatic synaptic scaling of AMPA receptors and consolidation of the synaptic plasticity (Bai et al., 2011). They could possibly play an important role in the neural recovery from the RABV infections.

Altogether, our results provide a broader view of the interplay between the RABV and the host. Virulent CVS-11 infection causes extensive inflammation, immune responses and nervous system dysfunction and induces many more altered genes than attenuated HEP-Flury infection. Once the infection is established, HEP-Flury, but not the CVS-11, can be effectively suppressed by innate immunity and cleared from the CNS prior to the induction of adaptive immunity. In the CVS-11 infected mouse brains, the expression of MHC class II and activation of CD4^+^ T cells were impaired, resulting in the failure to stimulate the VNAs and revealing a mechanism of immune suppression of the pathogenic RABVs. Genomic transcriptional analysis is an effective technique but not enough on its own to define the critical causes and effects of virus-host interactions. Extensive studies in different regulatory levels, using different RABV strains and inoculation routes are warranted in future investigations.

## Author contributions

Conceived and designed the experiments: YL, XG, DZ. Performed the experiments: DZ, FH, HG, BZ, FW, JL, YY, QT. Analyzed the data: YL, DZ, SB, CJ, HF, JC. Contributed reagents/materials/analysis tools: XG, YL. Wrote the paper: YL, DZ.

## Funding

This work was supported by grants from the National Natural Science Foundation of China (No. 31101842), Science and Technology Planning Project of Guangdong Province (No. 2014A020214006 and 2015A020209100) and Foundation for Fostering of Outstanding Young Teachers in Higher Education Institutions of Guangdong Province (No. yq2014029).

### Conflict of interest statement

The authors declare that the research was conducted in the absence of any commercial or financial relationships that could be construed as a potential conflict of interest.
